# FastGCN: A GPU Accelerated Tool for Fast Gene Co-Expression Networks

**DOI:** 10.1371/journal.pone.0116776

**Published:** 2015-01-20

**Authors:** Meimei Liang, Futao Zhang, Gulei Jin, Jun Zhu

**Affiliations:** Institute of Bioinformatics, Zhejiang University, Hangzhou, Zhejiang, RP China, 310058; The University of Hong Kong, HONG KONG

## Abstract

Gene co-expression networks comprise one type of valuable biological networks. Many methods and tools have been published to construct gene co-expression networks; however, most of these tools and methods are inconvenient and time consuming for large datasets. We have developed a user-friendly, accelerated and optimized tool for constructing gene co-expression networks that can fully harness the parallel nature of GPU (Graphic Processing Unit) architectures. Genetic entropies were exploited to filter out genes with no or small expression changes in the raw data preprocessing step. Pearson correlation coefficients were then calculated. After that, we normalized these coefficients and employed the False Discovery Rate to control the multiple tests. At last, modules identification was conducted to construct the co-expression networks. All of these calculations were implemented on a GPU. We also compressed the coefficient matrix to save space. We compared the performance of the GPU implementation with those of multi-core CPU implementations with 16 CPU threads, single-thread C/C++ implementation and single-thread R implementation. Our results show that GPU implementation largely outperforms single-thread C/C++ implementation and single-thread R implementation, and GPU implementation outperforms multi-core CPU implementation when the number of genes increases. With the test dataset containing 16,000 genes and 590 individuals, we can achieve greater than 63 times the speed using a GPU implementation compared with a single-thread R implementation when 50 percent of genes were filtered out and about 80 times the speed when no genes were filtered out.

## Introduction

Gene co-expression analysis provides a comprehensive way to identify the interactions between and the functions of certain genes. Additionally, co-expression analysis is an important step in bioinformatics analyses of gene expression data [1]. Currently, the majority of gene expression data are generated from microarray and RNA-seq methods. Microarray technologies have been widely used over the last few decades. With the emergence of high-throughput next-generation sequencing technologies, RNA-seq technology has been introduced. RNA-seq has several advantages over microarray technologies and may fully replace microarrays when several issues are overcome [2–4].

Gene co-expression networks comprise one type of valuable biological networks. One network can cover all human genes [5]. In gene co-expression networks, a gene is represented by vertexes and the edges that correspond to the pairwise correlations between expressions. Many methods have been published to construct gene co-expression networks [6–9]. The Pearson correlation coefficient is widely used. However, this method requires a substantial amount of time and space for large datasets [10–12]. Recently, Graphics Processing Units (GPUs) provide a promising tool to process tasks in parallel. GPUs are designed to have hundreds or even thousands of cores with small caches and logic control units. Therefore, the nature of GPU architecture makes it a powerful device for parallel computing rather than for data caching or branch control. The gene co-expression analysis for Pearson correlation coefficient computing is pairwise and easy to divide into small tasks. These tasks are independent and can be assigned to many-core GPUs for parallel processing. In the future, GPU computing should be used to construct the co-expression networks for large datasets [7].

CUDA (Compute United Device Architecture) C is a C-extended language developed by NVIDIA. This language is used to develop programs that can run on CUDA GPUs. The work pattern of GPU computing is SIMT (Single Instruction Multiple Thread). The code segment that starts a GPU is called Kernel. When Kernel runs, the thread space should be defined. The GPU threads are organized in Blocks. The thread number in Block is limited. No more than three dimensions are available for address space in Block. In an identical manner, Blocks are organized into a Grid, for which the address space should have no more than three dimensions. When the GPU starts computing, Blocks are assigned to SMX (prior to Kepler architecture, SMX was called SM) and running in a SIMT pattern. The number of running Blocks in SM depends on the Register number that one thread needs and the size of SMEM (Shared Memory). Threads should be busy for as much time as possible. The Block size and the resources that each thread claims leverage the entire resource.

The variables defined in Kernel are allocated in Registers automatically. Several shared variables or data structures are allocated in SMEM. SMEM and Registers are in the GPU chip; therefore, these two components have high access bandwidth. Other data are allocated in GMEM (Global Memory). To hide the GPU latency, the number of running warps (32 threads a warp) on the SM (Stream Multiprocessor) should be as many as possible. The GPU thread space is created by the syntax ⋘ grid, block⋙. In general, the size of the grid should be at least three times the number of SM. Moreover, there should be more than four warps in a Block.

In recent years, several tools and methods were introduced to harness the GPU power for increasing processing speeds in gene expression network analyses [13–17]. Here we propose a computationally efficient tool, FastGCN, to construct gene co-expression networks. The analysis procedure mainly contains four steps: preprocessing the input data with genetic information entropy, computing the Pearson correlation coefficient for the gene pair, transforming the coefficients to a normal distribution and identifying modules. GPU computing technology was exploited in all four steps. A sparse matrix compression was used in the correlation coefficient matrix. Our method outperformed the traditional gene co-expression analysis methods. This method is a powerful tool to assist the construction of gene co-expression networks. The source code and the executable binaries for this software are available at https://github.com/DrLiang/FastGCN.

## Methods

### Data Preprocessing with Genetic Information Entropy

Information entropy was proposed by Shannon [18] and has been widely used in industry to measure the uncertainty of a random variable. After the initial description, information entropy was heavily used in the life sciences [19–26]. In the present study, we used genetic information entropy to reduce the input data. For one dataset with *n* individuals and *m* genes, the genetic information entropy of the *i*-th gene can be defined as the following:

$$H_{i}=-\sum_{j=1}^{n}p_{ij}\log p_{ij}$$ (1)

where $p_{ij}=\frac{\left|x_{ij}\right|}{\sum_{k=1}^{n}\left|x_{ik}\right|}$, *x_ij_* is the expression abundance of the *i*-th gene of the *j*-th individual. When *p_ij_* reaches an equilibrium distribution (the *i*-th gene has no differential expression among the individuals), the genetic information entropy *H_i_* attains its maximum value. Therefore, we can filter a portion of the non-differentially expressed genes using the genetic information entropy.

### Pearson Correlation Analysis

For two genes *x* and *y*, the Pearson correlation coefficient is the following:

$$\rho_{xy}=(\sum_{i=1}^{n}x_{i}y_{i}-(\sum_{i=1}^{n}x_{i}\sum_{i=1}^{n}y_{i})/n)/\sqrt{\left(\sum_{i=1}^{n}x_{i}^{2}-{\left(\sum_{i=1}^{n}x_{i}\right)}^{2}/n)(\sum_{i=1}^{n}y_{i}^{2}-{\left(\sum_{i=1}^{n}y_{i}\right)}^{2}/n\right)}$$ (2)

where *x_i_* and *y_i_* are the expression abundances of genes *x* and *y* of the *i*-th individual, respectively.

### Normalization of Pearson Correlation Coefficients

For *k* genes, we are able to write *t* = *k*(*k* – 1) / 2 for the Pearson correlation coefficients. We normalized these coefficients to a test statistic, *z*, to test for the co-expression between two genes,

$$z_{i}=(\rho_{i}-\bar{\rho })/S$$ (3)

Where $S=\sqrt{\sum_{i=1}^{k}{\left(\rho_{i}-\bar{\rho }\right)}^{2}/(k-1)}$ is the standard deviation and $\bar{\rho }$ is an overall mean. Therefore, *z* is asymptotically distributed in a standard normal distribution. We can then transform the *z*-value to a P-value. Finally, we used the False Discovery Rate to control the multiple tests.

### Module Identification

With the normalized *z* as the evaluation criteria, we can easily transform coefficient matrix to adjacency matrix. The adjacency function is defined as:

$$a_{ij}=signum(q_{ij},\tau )=\{\begin{array}{c} \begin{array}{cc} 1 & q_{ij}\leq \tau \end{array}\\ \begin{array}{cc} 0 & q_{ij}>\tau \end{array} \end{array}$$ (4)

where *q_ij_* is the *q*-value for False Discovery Rate control. *τ* is threshold parameter. When the adjacency matrix is defined, the node connectivity can be got by:

$$c_{i}=\sum_{j}a_{ij}$$ (5)

To select the relevant genes and reduce the computational burden, the genes with *c_i_* = 0 are removed.

In gene co-expression network, modules are always defined as clusters of highly correlated genes [8,27–29]. We implemented module identification analysis based on topological overlap measure with a node similarity clustering method. The similarity matrix was defined as:

$$s_{ij}=\frac{h_{ij}+a_{ij}}{c_{i}+c_{j}-h_{ij}-a_{ij}}$$ (6)

where $h_{ij}=\sum_{u}a_{iu}a_{uj}$ is the number of shared nodes that connect to both node *i* and node *j*. *c_i_* and *c_j_* are the connectivity of node *i* and node *j*, respectively. The similarity matrix is also symmetric. These similar genes can form hot blocks which can be identified as modules along the diagonal.

## Implementation

We implemented the algorithms of these four steps using GPU technology with CUDA. The GPU works in a SIMT manner. When the GPU issues one instruction, many threads initiate on different data. These algorithms are able to exploit the parallel GPU computing power because each task was independent and easily assigned to one GPU thread.

### Data preprocessing on the GPU

For *m* genes (or exons) and *n* individuals in the raw input data, we created *m* GPU threads to compute the genetic information entropy. One entropy task was computed by one GPU thread. Using the traditional method, we should copy the data from the host memory to the GPU device memory to perform GPU computing. However, this method has two shortcomings in gene co-expression analysis. First, the raw data can be large but the GPU device memory is relatively small. Occasionally, the GPU device memory cannot accommodate all the input data, intermediate data and the result data. Second, transporting data from host memory to the GPU device memory belongs to I/O operations. I/O operations are inherently slow. To improve the performance, we should reduce the I/O operations as much as possible. To meet these challenges, Zero-copy technology was exploited. Zero-copy technology allows the GPU to use page-locked host memory. The GPU can directly access this part of the host memory. However, page-locked host memory is not page-able, and with overuse over a long time, this process can decrease the performance of the entire system. The asymptotic time complexity of entropy computation is *O*(*mn*). With GPU computing, this procedure can finish in a short time. Then, the occupied page-locked memory can be released. S1a Fig. shows the GPU architecture for the genetic information entropy computation. After determining the entropies from the GPU, the CPU took the responsibility of filtering the genes accordingly.

### Pearson Correlation Analysis on the GPU

The Pearson correlation analysis is a pairwise analysis. For *k* genes remaining after data preprocessing, *j* = *k*(*k* – 1) / 2 coefficients should be computed. The asymptotic time complexity of Pearson correlation analysis is *O*(*nk* ^2^). This analysis is the most time consuming step in the entire analysis. Generally, the coefficients are stored in a *k*×*k* strictly lower triangular matrix or upper triangular matrix. To save space, we used a packed matrix storage model. The packed matrix stores only the coefficients in a vector. To minimize the latency and improve the performance, as many GPU threads should be created as possible. We created *j* GPU threads. Each thread computed the coefficient of one gene pair. To implement this step, the gene index must be calculated by the GPU thread identification; therefore, we mapped the one-dimensional GPU thread space onto a two-dimensional input matrix space and one-dimensional result vector space. The mapping relationship is shown in S2a Fig. The GPU threads read the data from page-locked memory and stored the results in the GPU device memory. Then, the page-locked memory can be freed. S1b Fig. shows this pattern.

### Normalization of the GPU

We created *j* GPU threads in this normalization. One thread normalized one coefficient. The standard deviation in Equation 3 was shared between all GPU threads. Having each thread calculate the standard deviation separately is not an efficient method. Therefore, we used the summation reduction to determine the standard deviation of the threads cooperatively (S2b Fig.). The first half of the threads was used to sum the two coefficients. Then, the active threads were used at a half of the last step, and the summation was repeated. When the active threads were reduced to one thread, we achieved the final result. In the GPU memory hierarchy, shared memory is on-chip memory. On-chip memory is much faster than GPU device memory. Therefore, we used shared memory to achieve a high performance. However, if two or more threads request access to the identical memory bank when using shared memory, a bank conflict occurs. The access would then be serialized. To avoid bank conflict, we used sequential addressing. In this step, GPU threads are not independent from one another. The threads should be synchronized. To ensure the correct result, several barriers should be added to control the synchronization. This architecture is shown in S1c Fig.

### Module Identification on GPU

We implemented module identification with three GPU kernels. We also assume there are *k* genes. First, *k* GPU threads were created to get the adjacency matrix and the node connectivity vector. Then, we used CUBLAS, which is a CUDA library of basic linear algebra subroutines to calculate the matrix *h* defined in Equation 6. Finally, we used *k*×*k* GPU threads in two-dimensional thread space to compute the similarity matrix. We plotted S1d Fig. showing this architecture.

## Results

**Figure 1:**
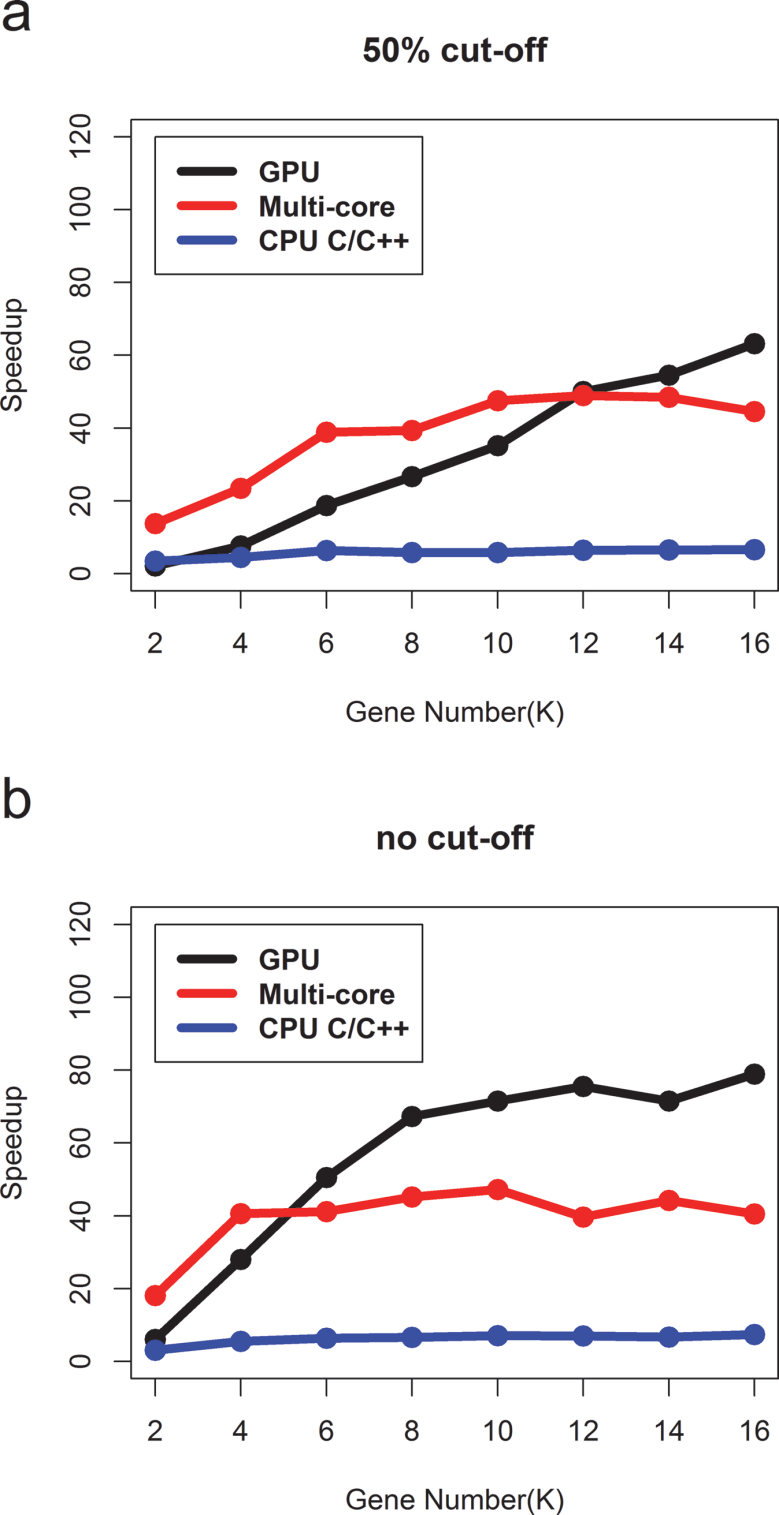
Curves of speedups against Single-thread R CPU implementation. (a) Speedup curves when 50% genes were filtered out at data preprocessing stage. (b) Speedup curves when no gene was filtered out at data preprocessing stage.

We have developed four versions of our tool FastGCN: GPU version, Multi-core CPU version, Single-thread CPU version with C/C++ and Single-thread CPU version in R. The version using the R language was highly optimized; we eliminated the explicit loops in all the computing procedures. To evaluate the performance of FastGCN for co-expression network inference, we used the GPU workstation consisting of a NVIDIA Tesla K20c card running on an Intel Xeon E5-2690 (16 CPU cores) with a clock rate of 2.90GHz using 256GB DDR3 host memory. We conducted several experiments using different datasets to compare the performance of these four versions. These datasets are extracted from the expression data covering Breast Invasive Cancer (BRCA) from TCGA [30] (https://tcga-data.nci.nih.gov/tcga/) which contains 17,814 genes and 590 individuals. Each dataset contains 590 individuals but has a different number of genes. We implemented two test groups: one with fifty percent of gene cutoff at the data preprocessing stage and the other with no gene cutoff. We tested the multi-core CPU version on 16 CPU threads running on 16 CPU cores. The results of this comparison are summarized in Table 1 and Table 2, respectively. This comparison showed the obtained running obtained as the mean running time of 1000 simulations. We calculated the running time from entropy preprocessing, the Pearson correlation coefficients calculated using the z-score normalization and module identification whereas excluding the input procedure and the output procedure because we only compared the computational performance of this tool. From Table 1, we can see that by using the GPU, the entire computational procedure of the dataset with 16,000 genes finished in about 2 seconds. However, 134.1 seconds were required for the single-thread R version, and 20.369 seconds were required for the single-thread C/C++ version. Therefore, the GPU implementation was approximately 63 times faster than the traditional serial CPU implementation in the R language and approximately 10 times faster than the CPU implementation with the C/C++ language. From Table 2, the GPU version can achieve speed increases of almost 80 times that of the single-thread R version. WGCNA[8] is a promising R package for correlation network analysis. The performance of WGCNA was better than the single-thread R implementation but worse than others. For the sake of immediacy, we also plotted Fig. 1A and Fig. 1B to show the speedup curves. These curves show that when the dataset is small, the multi-core version running on 16 CPU threads outperforms the other processes. However, as the size of dataset increases, the GPU version can surpass the multi-core version in terms of performance.

To illustrate the application of FastGCN in the construction of a co-expression network of gene expression data, FastGCN was applied to the Breast Invasive Cancer (BRCA) data from TCGA. The genes in the HTLV-I infection pathway from the KEGG database (http://www.genome.jp/kegg/pathway.html) were included in the analysis.

**Table 1:** Running time (in seconds) for the GPU implementation, Multi-core CPU implementation, Single-thread C/C++ implementation and Single-thread R implementation when 50% of the genes were filtered out during the data preprocessing stage (individual number = 590).

**Gene Number**	**GPU**	**Multi-core CPU**	**Single-thread C/C++**	**Single-thread WGCNA**	**Single-thread R**
**2k**	0.624	0.094	0.375	1.622	1.295
**4k**	0.671	0.218	1.138	5.020	5.101
**6k**	0.811	0.39	2.403	14.102	15.163
**8k**	0.967	0.655	4.447	25.032	25.740
**10k**	1.202	0.889	7.301	41.769	42.260
**12k**	1.388	1.419	10.811	69.685	69.420
**14k**	1.747	1.965	14.633	94.731	95.220
**16k**	2.122	3.010	20.369	130.627	134.100

Multi-core CPU version ran on 16 CPU threads running on 16 CPU cores.

**Table 2:** Running time (in seconds) for the GPU implementation, Multi-core CPU implementation, Single-thread C/C++ implementation and Single-thread R implementation when no genes were filtered out during data preprocessing stage (individual number = 590).

**Gene Number**	**GPU**	**Multi-core CPU**	**Single-thread C/C++**	**Single-thread WGCNA**	**Single-thread R**
**2k**	0.655	0.218	1.295	4.529	3.947
**4k**	0.858	0.592	4.384	24.250	24.020
**6k**	1.295	1.591	10.311	63.971	65.400
**8k**	1.950	2.902	19.906	127.037	131.148
**10k**	3.089	4.68	31.31	220.112	221.040
**12k**	4.321	8.236	46.863	322.876	326.34
**14k**	6.771	10.968	72.758	480.106	484.56
**16k**	8.003	15.600	85.597	618.084	632.04

Multi-core CPU version ran on 16 CPU threads running on 16 CPU cores.

**Figure 2:**
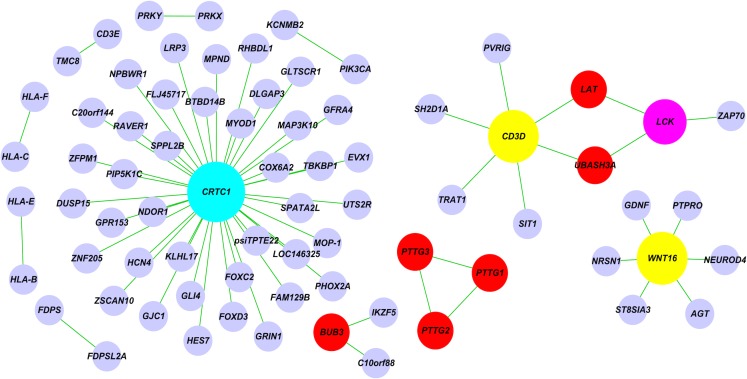
The co-expression network reconstructed by FastGCN using BRCA expression data with HTLV-I infection pathway.

The co-expression network constructed by FastGCN is shown in Fig. 2. We identified three hub genes that are heavily connected: *CRTC1, CD3D* and *WNT16*. These hub genes were of functional importance. These hub genes have been reported to be associated with mortality [31–34]. Previous studies have also reported that *CD3D, LCK* and *ZAP70* are relatively expressed [35].

## Discussion

Numerous tools have been proposed for analyzing gene co-expression. However, the majority of these tools are not popular because they are inconvenient to use and have prohibitive computational time because of the extremely large number of tests. To address these problems, we have presented a user-friendly, accelerated and optimized tool for gene co-expression analysis. We used a novel entropy based method for data preprocessing. According to the entropy of each gene, we can make the reduction to cutoff genes with no or small expression changes. From Table 1 and Table 2, this method was shown to alleviate the computational burden. Because the gene number was reduced, the multiple tests could also be partially controlled.

Taking advantage of the parallel nature of multi-core GPUs, the parallel version of this tool can highly reduce the computational time compared to the traditional single-thread version. GPUs and CPUs have different architectures. GPUs are designed to have a higher memory bandwidth and more transistors for data parallel processing but less ability for data caching and branch control than CPUs[36]. Therefore, GPU computing is suitable for a program that has few branches and is independently executed on many data elements. We accelerated the entropy calculation, correlation calculation, z-score transformation and module identification on the GPU because these four procedures are independent and parallel and have minimal branches. Using the datasets illustrated in Table 1 and Table 2, the computation of these four procedures can be completed in a few seconds on a GPU. Additionally, we achieved promising speedups over a single-thread R or C/C++ implementation on a CPU. For example, using a dataset with 16,000 genes and 590 individuals, the entire computational procedure can be finished about 2 seconds on a GPU, which is 63 times the speed of the R implementation and 10 times the speed of the C/C++ implementation on a CPU (when we filter 50 percent of the genes in the data preprocessing step). If no genes were filtered, then the entropy calculation would not be invoked. Using the identical dataset, the computational procedure finished in 8 seconds on a GPU. This completion time was approximately 80 times the speed of the R implementation and more than 10 times the speed of the C/C++ implementation on a CPU.

The GPU cannot reach its maximum potential with small datasets. The multi-core program is faster than the GPU program. However, the GPU overtakes the multi-core CPU when the dataset is greater than 6000 without a gene filter or than 12,000 genes when half of the genes are filtered. The reasons for this higher efficiency are mainly because of 1) the overhead of the schedule of the GPU kernels, 2) the overhead of the data transfer from the GPU memory to host memory, 3) and small GPU thread space created for a small dataset. More precisely, a GPU can provide a benefit only when the reduction in the computing time on the GPU exceeds the cost of the overhead. To improve the performance of the GPU, as many GPU threads should be created as possible to offset the overhead. For a small dataset, the computational complexity may not be large enough to offset the cost of the overhead. As the dataset increases, the computational workloads will increase and additional GPU threads will be created. Therefore, the GPU performance improves and eventually overcomes the multi-core CPU as shown in Fig. 1. For analyzing small datasets, we also provide the multi-core version in our source code packages. To achieve high performance on the GPU, FastGCN was designed to avoid branches, to exploit matrix compression and to store gene expression data in major columns to coalesce GPU global memory. The data fetched by one global memory access can serve as many threads as possible in a warp.

The main purpose of this article is to emphasize that the entropy reduction method and the new GPU parallel computing technology can collaboratively contribute to gene co-expression analysis. Therefore, we hope the tool we developed will be wildly used in gene expression analyses.

## Supporting Information

S1 Fig GPU architectures implemented in FastGCN. (a) GPU architecture of genetic information entropy computation. (b) GPU architecture of Pearson Correlation Coefficients computation. (c) GPU architecture of Z-score transformation. (d) GPU architecture of modules identification.
(TIF)

S2 Fig Optimization strategies in GPU implementation. (a) mapping relationship among one-dimensional GPU thread space, two-dimensional input matrix space and one-dimensional coefficient vector space. (b) Summation reduction of the Pearson Correlation Coefficients in GPU.
(TIF)
