# Supplementary figures and images for: FastGCN: A GPU Accelerated Tool for Fast Gene Co-Expression Networks
Source: PLoS One. 2015 Jan 20;10(1):e0116776. doi: 10.1371/journal.pone.0116776 (PMC4300192; doi:10.1371/journal.pone.0116776)

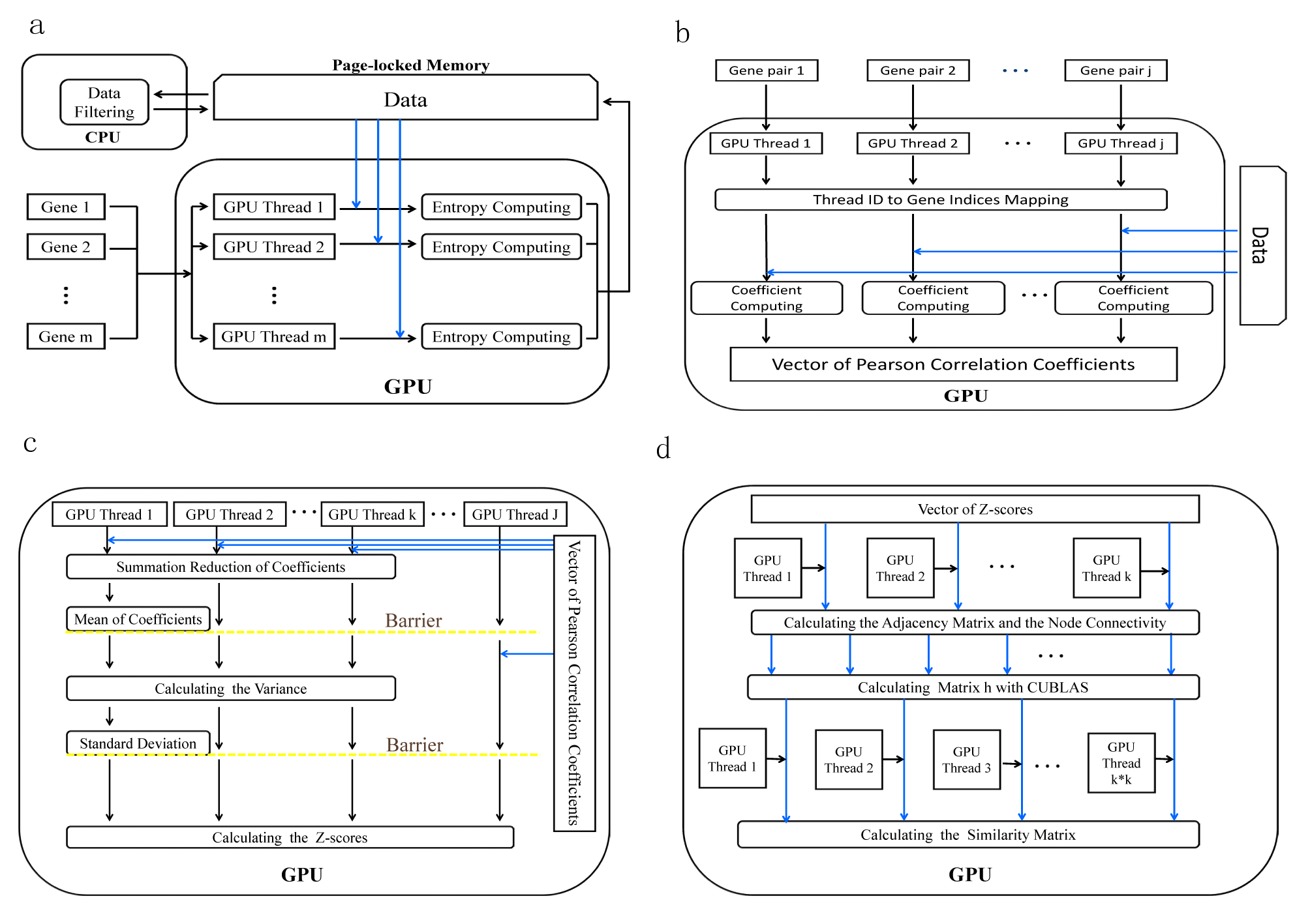

Supplement: S1 Fig — (a) GPU architecture of genetic information entropy computation. (b) GPU architecture of Pearson Correlation Coefficients computation. (c) GPU architecture of Z-score transformation. (d) GPU architecture of modules identification. (TIF) [file pone.0116776.s001.tif]

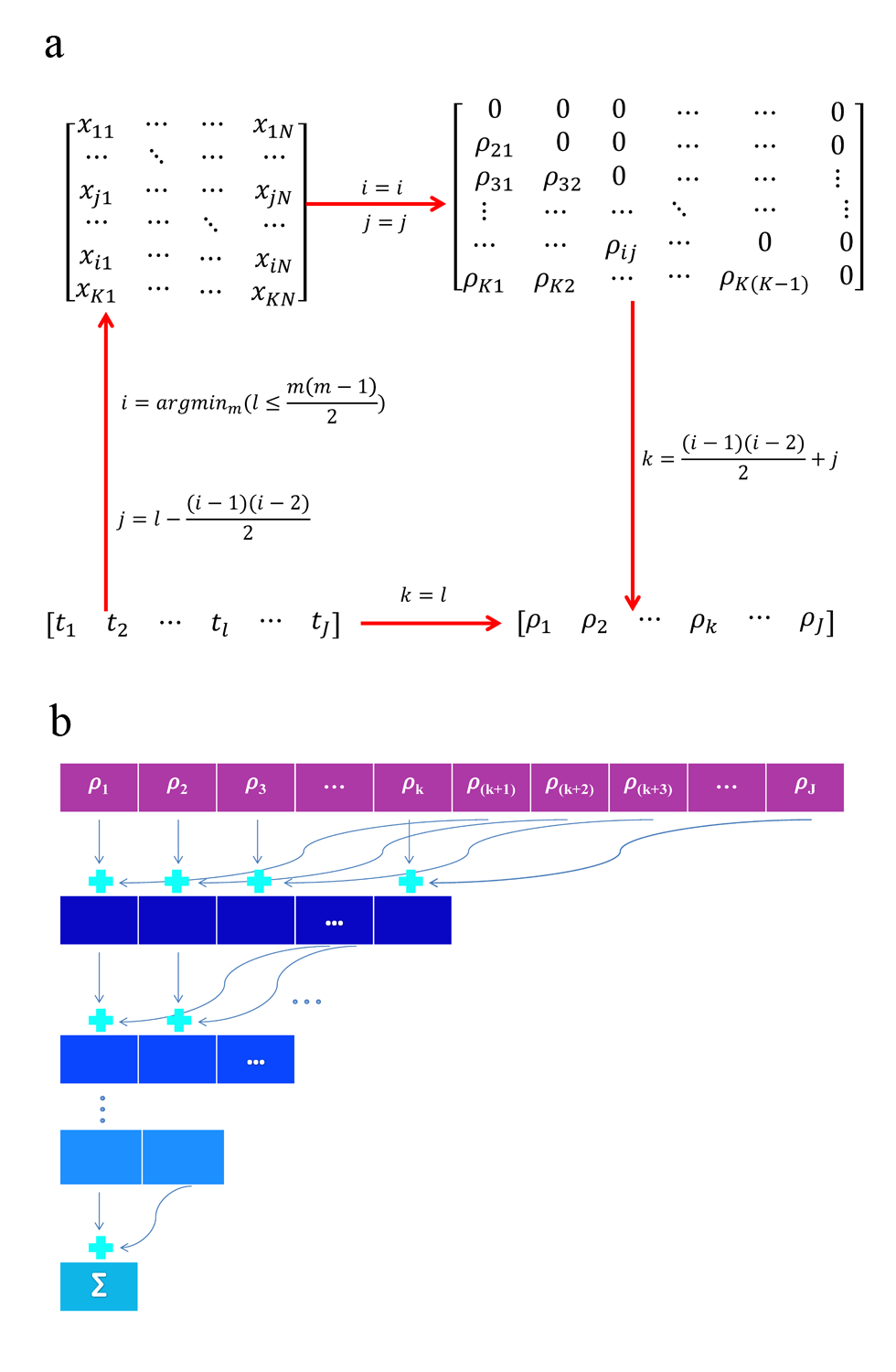

Supplement: S2 Fig — (a) mapping relationship among one-dimensional GPU thread space, two-dimensional input matrix space and one-dimensional coefficient vector space. (b) Summation reduction of the Pearson Correlation Coefficients in GPU. (TIF) [file pone.0116776.s002.tif]
